# Multi-channel convolutional neural network architectures for thyroid cancer detection

**DOI:** 10.1371/journal.pone.0262128

**Published:** 2022-01-21

**Authors:** Xinyu Zhang, Vincent C. S. Lee, Jia Rong, Feng Liu, Haoyu Kong

**Affiliations:** 1 Department of Data Science and AI/Faculty of IT, Monash University, Melbourne, Victoria, Australia; 2 Department of Human-Centred Computing/Faculty of IT, Monash University, Melbourne, Victoria, Australia; 3 West China Hospital of Sichuan University, Chengdu City, Sichuan Province, China; Vietnam National University, VIET NAM

## Abstract

Early detection of malignant thyroid nodules leading to patient-specific treatments can reduce morbidity and mortality rates. Currently, thyroid specialists use medical images to diagnose then follow the treatment protocols, which have limitations due to unreliable human false-positive diagnostic rates. With the emergence of deep learning, advances in computer-aided diagnosis techniques have yielded promising earlier detection and prediction accuracy; however, clinicians’ adoption is far lacking. The present study adopts Xception neural network as the base structure and designs a practical framework, which comprises three adaptable multi-channel architectures that were positively evaluated using real-world data sets. The proposed architectures outperform existing statistical and machine learning techniques and reached a diagnostic accuracy rate of 0.989 with ultrasound images and 0.975 with computed tomography scans through the single input dual-channel architecture. Moreover, the patient-specific design was implemented for thyroid cancer detection and has obtained an accuracy of 0.95 for double inputs dual-channel architecture and 0.94 for four-channel architecture. Our evaluation suggests that ultrasound images and computed tomography (CT) scans yield comparable diagnostic results through computer-aided diagnosis applications. With ultrasound images obtained slightly higher results, CT, on the other hand, can achieve the patient-specific diagnostic design. Besides, with the proposed framework, clinicians can select the best fitting architecture when making decisions regarding a thyroid cancer diagnosis. The proposed framework also incorporates interpretable results as evidence, which potentially improves clinicians’ trust and hence their adoption of the computer-aided diagnosis techniques proposed with increased efficiency and accuracy.

## Introduction

The thyroid, a butterfly-shaped endocrine gland, is crucial in the human body to control heart rate, blood pressure, and body temperature [1]. Since the diagnostic instances of malignant thyroid nodules have continuously increased in the past 50 years [2], this critical organ has grabbed quite a lot of attention worldwide. Statistics suggest that more than 300 million people were suffering from thyroid disease in 2018 [3], and the number is still rising today. As the most commonly seen disease for men in the age group 30 to 39, thyroid cancer was even more prevalent in female groups with three times more possibilities of diagnosis than males [4]. Despite the continuously increasing thyroid disease instances, its diagnostic procedure in the clinics has not been improved since the 20th century [5].

Typical thyroid disease includes functional and neoplastic, and they are diagnosed through manual processes. Thyroid function examination is required to detect thyroid functional disease (i.e., hypothyroidism and hyperthyroidism). Two main hormones were produced from thyroid glands: triiodothyronine (T3) and thyroxine (T4) [6]. Besides T3 and T4, free T3 (FT3) and free T4 (FT4), along with thyroid-stimulating hormones (TSH), compose the thyroid function examination to diagnose hypothyroidism and hyperthyroidism [7]. Regarding thyroid neoplastic disease, which are thyroid nodules, clinicians divide them into benign nodules and malignant kinds (cancerous cells). Early detection and diagnosis of abnormal thyroid nodules can prevent cancer, leading to decreased morbidity and mortality rates.

Standardized thyroid nodule detection procedure requires medical imaging, such as computed tomography (CT), magnetic resonance imaging (MRI), radio-iodine scintigraphy, positron emission tomography (PET) scanning, as well as ultrasound images which are widely adopted tools for helping the diagnosis of thyroid disease [8]. Unfortunately, medical imaging cannot fully support differentiating between benign and malignant thyroid nodules; this is when fine-needle aspiration cytology (FNAC) occurs [9, 10].

FNAC is to get cells from thyroid nodules for pathological examinations, and it is even regarded as the golden standard for diagnosing thyroid cancer [11]. However, the FNAC diagnostic accuracy rates highly depend on the pathologists’ experience and expertise. Learning from that, around 30% FNAC results ended being not determined or non-diagnostic due to pathologists’ lack of experience [12]. Patients have to suffer from undergoing another FNAC or excisional biopsy to examine the abnormal nodules under this circumstance. Sometimes, patients would have already undergone unnecessary surgeries when identifying those nodules show no evidence of malignancy [13]. Accordingly, a novel way of detecting thyroid disease has emerged by drawing technical support from machine learning with medical imaging to distinguish between benign and malignant thyroid nodules.

Machine learning approaches, specifically deep learning techniques, have been utilized frequently in the clinical field, including predicting, diagnosing, and curing disease, known as computer-aided diagnosis (CAD) techniques. With thyroid disease, researchers tend to apply various statistical [14], and machine learning classification algorithms on diagnosing hypothyroidism and hyperthyroidism, such as k-nearest neighbor [15], linear discriminant analysis [16], decision tree [17] to name a few. Meanwhile, deep learning algorithms were often adopted for classifying thyroid nodules through medical images.

Researchers have previously put much effort into improving thyroid disease diagnostic accuracy rates by engaging with ultrasound images. Mostly, the classification tasks for malignant thyroid nodules detection using ultrasound images reached accuracy rates ranged from 72% to 92% in variety [6, 18–21], appearing to outperform inexperienced radiologists. Nevertheless, ultrasound images have limitations since they are susceptible to noises and speckles [22]. Overall, applying ultrasound images to classify individual abnormal nodules is inefficient and time-consuming, and relying on a single image modality to determine diagnostic risk stratification is not evident enough, leading to absent adoptions in clinical.

Besides ultrasounds, CT scans are always recommended prior to operations for evaluating central lymphatic metastasis. Existing literature shows that CT presented significantly higher accuracy than ultrasounds when detecting cancer metastasis by the clinical [23], suggesting it is also making benefit for cancer diagnosis. Nevertheless, studies on adopting CT scans for detecting abnormal thyroid glands are far limited.

Hence, we propose a multi-channel deep learning framework, taking Xception as the base structure, and designs three different architectures to detect thyroid cancer. Xception is the state-of-art deep learning convolutional neural network (CNN) that is more efficient with higher accuracy compared to VGG networks, Residual networks, and Inception networks [24]. Through the multi-channel framework, researchers can have explicit knowledge of comparing the two choices of medical imaging modalities (i.e., ultrasound and CT) regarding their effectiveness and model generalizability, leading to enhanced CAD adoptions in clinical.

Accordingly, this paper makes contributions in the following four main ways:

- To our knowledge, this paper is the first of its kind that adopts Xception, the current state-of-art CNN, on thyroid cancer detection tasks. Additionally, Xception was designed as the base structure for the multi-channel framework development, taking filter size selection into consideration for multi-channel CNN design for cancer detection is the novelty in this paper. Our work has the potential application for clinical use to release patients suffering from time-consuming and painful diagnostic procedures.
- We have compared the Xception model with ten other popular CNN models to prove the feasibility of selecting Xception as the base model. In addition, the proposed multi-channel architectures using Xception as base structure have also been evaluated through different medical image modalities. The results are interpretable for clinicians and suggest that Xception significantly increases diagnostic accuracy rates with efficiency.
- This paper also incorporates comparative analysis. For ultrasound images, comparisons were made between acquired hospital data sets and open access data sets to demonstrate the increased diagnostic accuracy comparing to existing literature studies. Comparisons were also made between ultrasound images and CT scans so that researchers can have a clear view of the decision-making regarding medical images’ choices. This work may extend the way we conduct researches, and potentially more types of medical image modalities can be incorporated for CAD techniques.
- Our work is highly reproducible since we have utilized open access data sets, and our architecture code implementation is available through GitHub.

## Related work

Thyroid disease includes functional (i.e., hypothyroidism, hyperthyroidism, and thyroiditis) and neoplastic (i.e., goiter, adenoma, and four types of malignant thyroid nodules. These four malignant types of nodules are formed as four kinds of thyroid cancer: papillary carcinoma, follicular carcinoma, anaplastic carcinoma, and medullary carcinoma [8]. A considerable number of studies have adopted CAD approaches to diagnose thyroid disease in the past decades, and this section provides an overview of the studies and elaborates on the research gaps.

### Machine learning techniques for thyroid disease detection

Based on previous studies, binary classification tasks were performed the most frequently. Among those studies, machine learning techniques have been adopted quite often on classifying between hypothyroidism and hyperthyroidism. For instance, [15] have proposed a hybrid decision support system for diagnosing thyroid disorder by combining linear discriminant analysis (LDA), k-nearest neighbors (KNN), and adaptive neuro-fuzzy inference system, and they have reached an accuracy of 98.5%. LDA was also adopted by [17], and based on his experiments, the hypothyroidism and hyperthyroidism classification accuracy rates reached 99.62%. Similarly, random forest (RF), support vector machine (SVM), and KNN were also applied separately with 98.5% accuracy rates obtained by the RF approach [25]. With the help of machine learning techniques, thyroid disorder can be efficiently detected [26–38].

Compare to diagnosing thyroid disorder, detecting thyroid cancer is another intense area for scholars to excavate. Classifying thyroid nodules into benign and malignant is a demanding research direction due to increased thyroid cancer instances. Many researchers have adopted machine learning techniques to extract various features from ultrasound images for thyroid cancer detection. The most commonly seen features used for classifying thyroid nodules through ultrasound images are nodule size, echogenicity, microcalcifications, margin, shape, contour, and vascularity [39]. [40] applied a Bayesian classifier and evaluates 49 nodules, resulting in obtaining the area under the curve value of 0.851. Other than the Bayesian classifier, the extreme learning machine (ELM) was applied with 203 nodules using ultrasound images and achieved an accuracy of 87.72% [21]. Besides these, SVM [39], KNN [12], LDA [41], and other machine learning algorithms have been proven to be effective in thyroid cancer diagnosis.

### Deep learning techniques for thyroid cancer detection

In the past few years, deep learning has become an intense subject in machine learning. Machine learning has brought irrefutable promising diagnostic accuracy rates for thyroid disease detection. Meanwhile, deep learning can select features from inputs automatically and results in increased diagnostic efficiency and accuracy. With the emergence of deep learning techniques, image classification is an impartible area that has been widely utilized in various fields, such as education, industry, and most importantly, the medical field. Furthermore, deep neural networks form a new way of making classifications and have been adopted by countless scholars to diagnose diversified diseases, like breast cancer [42, 43], melanoma disease [44], macular edema detection [45], and lung cancer [46, 47].

Artificial neural networks (ANN) and CNN are the two most commonly used and outstanding deep learning models for classifying thyroid nodules. Previous studies indicated that ANNs were accurate for distinguishing thyroid nodules between “benign” and “malignant” and have obtained accuracy rates around 82% [48, 49].

**Fig 1:**
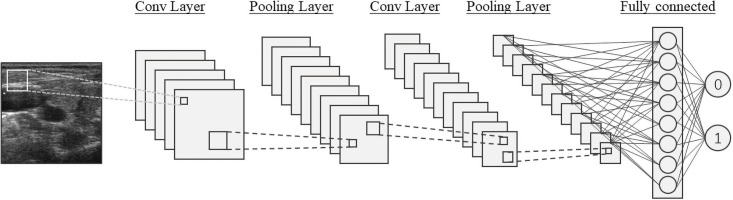
Typical single-channel CNN architecture.

Concerning CNN, Fig 1 presents a typical single-channel CNN architecture that consists of convolutional layers, pooling layers, and fully connected layers.

As for thyroid cancer detection tasks, scholars determined that CNN obtained record-breaking accuracy rates compared to ANN. For instance, [19] have proposed two CNNs to detect malignant thyroid nodules using ultrasound images, and the best accuracy rate reached 83.02%. Similar research was conducted by [20], 589 nodules were tested in three CNNs, and the best accuracy rate is 85%. Besides, with ultrasound images through the InceptionResnetV2 model and residual networks, 87.3% accuracy was reached by [22], 93.75% was achieved by [50].

Rather than applying ultrasound images for diagnosing thyroid disease, quite a few studies also adopted other medical images for abnormal thyroid nodules classification [51, 52]. Overall, CNN are significantly efficient in diagnosing thyroid disease through medical imaging [53–57].

### Multi-channel CNN for CAD design

Conventional CNN architectures, such as VGG models, are eminent in image classification tasks [58], while researchers raised a concern that such a model might not be the right choice for medical images since medical images are much simpler in structures compared to natural images [59]. With more and more advanced CNN architectures being designed, multi-channel CNN architectures were built and evaluated for sentiment analysis [60], high dynamic range imaging [61], or sentence relation classification [62], all showing ascendant performance. With the goal of increasing medical image classification performance in mind, researchers incorporate multi-channel designs in the medical field.

For instance, [63] designed a novel neural network called “LVSNet” for liver vessel segmentation. Within the designed network, they have introduced a multi-scale feature fusion block that evenly divides the features into 4 filter groups; those filter groups are then connected in hierarchical residual learning; in this way, the number of scales in one block can be enhanced. Similarly, [64] proposed a “Progressive Atrous Spatial Pyramid Pooling” (PASPP) block for chest CT image segmentation to detect coronavirus disease 2019 (COVID-19) through adopting different dilation rated convolutions to perceive features with various scales.

Additionally, [65] adopted magnetic resonance image (MRI) with multi-channel CNN to identify infants who have the risk of autism disease and obtained an accuracy of 0.724. Several other research studies accentuated that multi-channel CNN architectures demonstrate enhanced diagnostic performance for particular disease [66–68].

Among all the related literature studies, multi-channel CNN architecture designs are distinct depending on the tasks, and researchers tend to ignore the fairly strong association between filter size and CNN performance. Therefore, this research study proposes a multi-channel CNN architecture that renovates filter size selection while taking advantage of CT characteristics to drive a diagnosis of thyroid cancer.

### Overall research gaps

Studies on detecting malignant thyroid nodules are ample, yet those algorithms’ applications are still absent in the clinical field since they have some limitations. Thyroid nodules are widespread, and more than 50% adults have them, while 5% of the nodules turned out to be malignant, known as thyroid cancer [39]. Existing studies can help clinicians differentiate malignant thyroid nodules to a great extent, yet most models take time to classify nodules one by one, which is time-consuming and inefficient. Besides, thyroidectomy is expected for removing all malignant thyroid nodules rather than thyroid glands solely. Hence, classifying each nodule via ultrasounds is time-consuming, and locating malignant nodules during surgeries is impracticable, not to mention the sensitiveness of ultrasound images to speckle noises. Therefore, the implementation of binary classification tasks using ultrasound images is still challenging for clinical adoptions. Compared to ultrasound images, CT is an efficient tool for detecting abnormal thyroid glands with higher diagnostic accuracy rates [23], and it is always required before surgeries. Nevertheless, studies adopting CT for thyroid cancer detection are still far lacking.

In summary, clinicians usually need to test different CNN models for identifying the most appropriate one for classifying thyroid nodules [12]. With the emergence of various deep learning models, conventional CNN architectures might be outdated as they require more computational resources for generating deeper models in image classification tasks [69]; thus, Xception is currently the right choice due to its enhanced performance with efficiency [24]. Hence, this research study proposes a framework with adaptive multi-channel architecture that incorporates Xception structure. Regarding highlighting the use of different medical image modalities, a comparison between ultrasound images and CT scans was presented in this paper. The framework allows clinicians to choose the most suitable architecture for thyroid cancer detection, ignoring the input data set characteristics, selecting the output choices, and making it applicable to both balanced and imbalanced data sets.

## Methodology

This section presents and explains the proposed “multi-channel Xception-based thyroid cancer detection” (MXTCD) framework.

### The MXTCD framework

**Fig 2:**
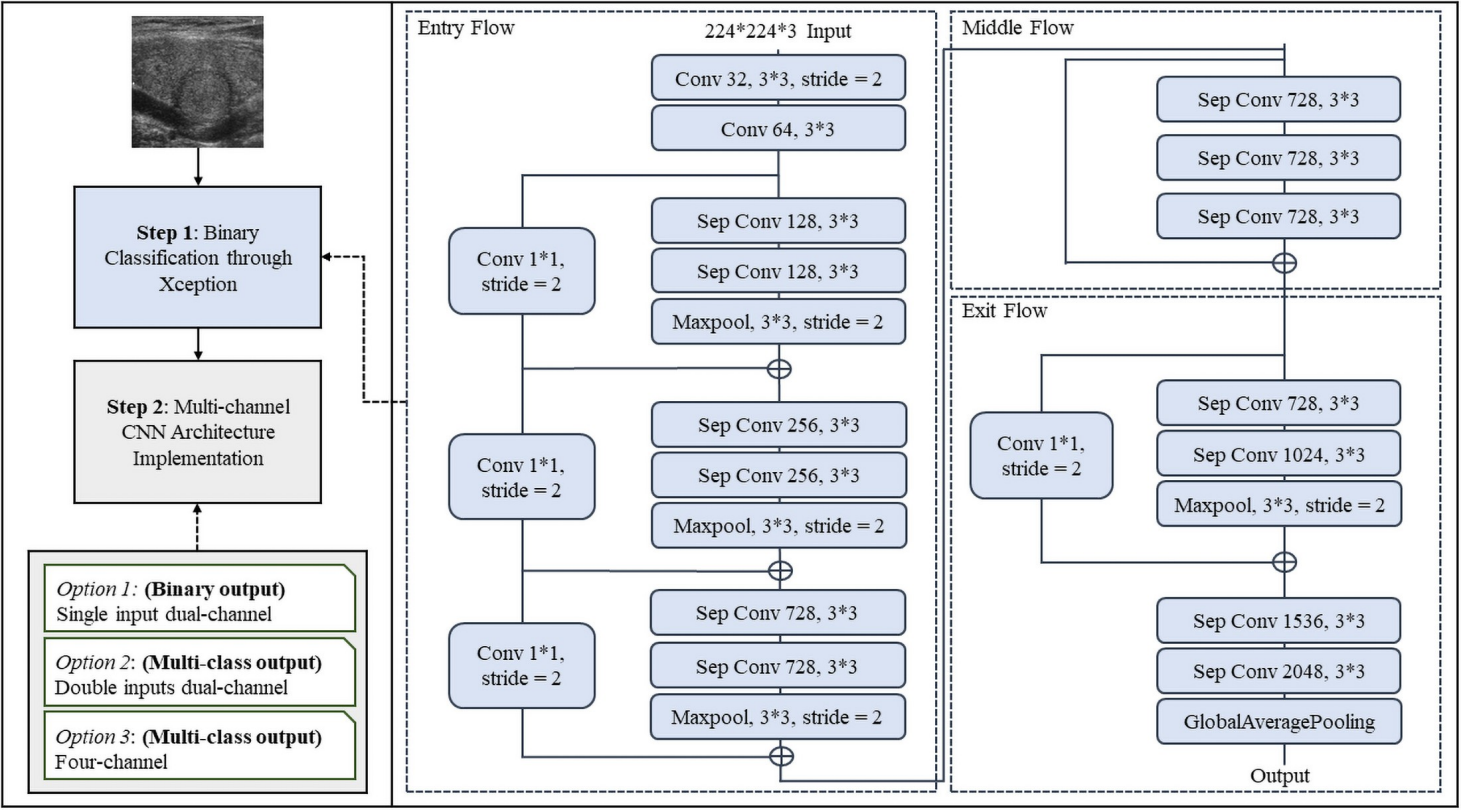
Multi-channel Xception-based thyroid cancer detection framework.

Based on the literature review, scholars have obtained satisfying diagnostic accuracy for thyroid cancer by applying various machine learning approaches. However, in reality, particularly for medical imaging research, data sets always have high complexity with different characteristics (e.g., balanced or imbalanced data, different image types, image quantity, and image qualities); with those uncertainties, no model best fits all types of data sets. Additionally, many pieces of research were conducted on transfer learning. Nevertheless, it is necessary to design a model for the specific disease. To support clinicians regarding thyroid disease diagnosis, here presents a multi-channel Xception-based framework with a flexible architecture (see Fig 2). The framework allows researchers to obtain the best accuracy rates ignoring the input characteristics’ differences (i.e., whether the data set is balanced or imbalanced) but optionally selecting the output features.

Common deep learning models utilized for thyroid nodules classification tasks are VGG models (e.g., VGG16 and VGG19) [13, 52], InceptionV3 [70], ResNet50 [55] to name a few. Each model has presented satisfactory diagnostic results for different input data sets, while this paper adopts the current state-of-the-art CNN model to detect thyroid cancer. In order to evaluate our choice of CNN selection, we have compared our base Xception model with three ResNet models (i.e., ResNet10, ResNet18, ResNet50), four VGG models (i.e., VGG8, VGG11, VGG16, VGG19), DenseNet121, InceptionV3, and InceptionResNetV2 models.

The MXTCD framework was designed and consisted of two main steps. Initially, **Step 1** indicates that we input medical images into the Xception model for pre-training purposes using binary classification tasks. This paper incorporates real-world CT scans and ultrasound images to present precise knowledge to the readers regarding the impact of medical image type selections on the detection results. Due to the different input features, we have trained and also fine-tuned the model separately for different data sets. In order to have a comprehensive comparison among all the selected models, the models were then evaluated based on their accuracy rates, *f*1 score, precision, recall, negative predictive value (npv), and running time.

**Step 2** applies the Xception-based multi-channel architectures for binary and multi-class classification tasks depending on the clinicians’ needs. Three optional architectures are proposed for selections: the single input dual-channel (SIDC), the double inputs dual-channel (DIDC), and the four-channel architectures. Researchers can obtain the enhanced accuracy rates for the specific input data set characteristics regarding thyroid cancer detection through those architectures.

### Xception

Xception, known as “Extreme Inception”, was inspired by Inception models and was firstly introduced by [24]. The Inception networks compute the convolutions of different filter sizes and the pooling layers in parallel and select the sequence of convolutions and pooling layers combination by itself [71]. [69] proposed the Inception models that are deeper in layers but with fewer parameters so that utilization of computational resources was improved. Then the Xception has all the inception modules designed as depth-wise separable convolutions [24]. The detailed architecture of the Xception model can be viewed in Fig 2.

Xception model maps cross-channel correlations from the input image and addresses spatial correlations of each output channel separately. Therefore, we intend to adopt the Xception model in this work to identify abnormal thyroid nodules from medical images since the model emphasizes spatial correlations of the inputted medical images to better locate and classify the lesion.

Besides, [24] applied Xception on ImageNet and had it compared to VGG16, ResNet152, and InceptionV3, Xception reached the best accuracy rates among the models and finished training and testing with the least running time obtained.

In the medical field, clinicians need to deal with a vast amount of complex medical images, making Xception the most appropriate choice since it can provide enhanced diagnostic accuracy and efficiency compared to traditional clinical diagnosis and conventional CNN models [24]. Our ablation evaluations also supported this statement. Therefore, this study selects Xception as the base structure for implementing the multi-channel architectures.

### Multi-channel CNN model

In this section, the three optional multi-channel architectures were explained based on their design and implementation.

#### Single input dual-channel CNN model

**Fig 3:**
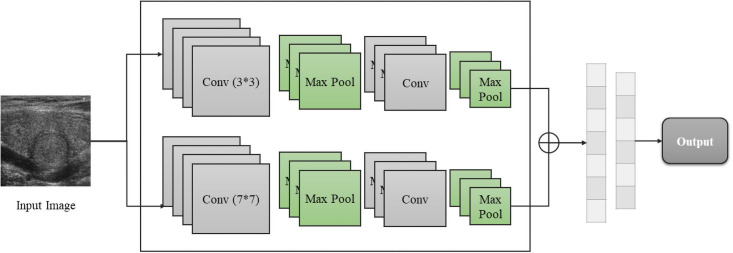
Single input dual-channel model.

We proposed three kinds of architectures for enhancing thyroid disease detection in this study, and the first option from Fig 2 is the Single Input Dual-channel CNN (SIDC). Medical images are in different formats and qualities, and sometimes researchers rely on a single type of medical image for making decisions through CAD techniques. In order to obtain a promising diagnostic result for this situation, the SIDC model is designed and presented in Fig 3.

Conventional CNN models usually involve a single channel (see Fig 1). Here, we present two channels for diagnostic accuracy enhancements. Given an input dataset *X*, after encoding the images, each image from *X* will go through two convolutional channels simultaneously with different filter sizes 3 and 7, then following max-pooling layers until it finishes traveling through the Xception architecture and produce two feature maps. Finally, the generated two feature maps by two convolutional channels will be concatenated and together sent to the fully connected layers and produce a classification output. Filter size, as a hyper-parameter, has been proven able to significantly affect the performance of any CNN model [72]. We have evaluated our filter size selection for this research. Besides, two different convolutional kernels mean different receptive fields, concatenation makes more spatial information to be maintained through the dual-channel architecture [73]. The main advantage of applying the dual-channel architecture is:

- With the SIDC architecture, enlarged receptive fields can lead to more valuable information be obtained from the original input image; thus, increased classification accuracy rates can be achieved.

In the SIDC architecture, there is only one input stream. Therefore, only one image can be applied and classified each time. By looping through dual-channel architecture, different filter sizes can automatically learn different features from the input images. Specifically, smaller filter sizes will learn detailed textures such as edges, while larger filter sizes will learn more abstract features. In this case, the feature map was calculated with Eq 1 where *Y*_*k*_ denotes the *k*th output feature map size, *f*(.) is the activation function, *W*_*k*_ is the *k*th feature map in the corresponding convolutional filter deciding by the kernel size, and *X* is the encoded input image.

$$\begin{array}{c} {\text{Y}}_{\text{k}}=f\left({\text{W}}_{\text{k}}*X\right) \end{array}$$ (1)

Accordingly, the follow-up feature map was calculated depending on the filter and stride size using Eq 2. Here, *F* represents the size of the generated feature map, *n*_*h*_ and *n*_*w*_ stand for the image’s height and width, *f* is the corresponding filter size, *s* is the stride step, and *n*_*c*_ is the number of channels the image has.

$$\begin{array}{c} F=\left(\frac{{\text{n}}_{\text{h}}-f}{s}+1\right)*\left(\frac{{\text{n}}_{\text{w}}-f}{s}+1\right)*n_{c} \end{array}$$ (2)

Additionally, both convolutional channels are followed by max-pooling procedures, which select the most significant element in each receptive field and are activated using rectified linear unit (ReLU) function [71, 74].

$$\begin{array}{c} ReLU\left(x\right)=\{\begin{array}{cc} x, & \text{if}\;x\geq 0.\\ 0, & \text{otherwise}. \end{array} \end{array}$$ (3)

Also, depending on the different CNN architecture, we suggest concatenating the feature maps from both channels, following a fully connected layer with a softmax operator used for interpreting and classifying features, as the same as the typical CNN architecture [75]. With the SIDC architecture, the performance of the thyroid cancer detection task can be enhanced.

#### Double inputs dual-channel CNN model

**Fig 4:**
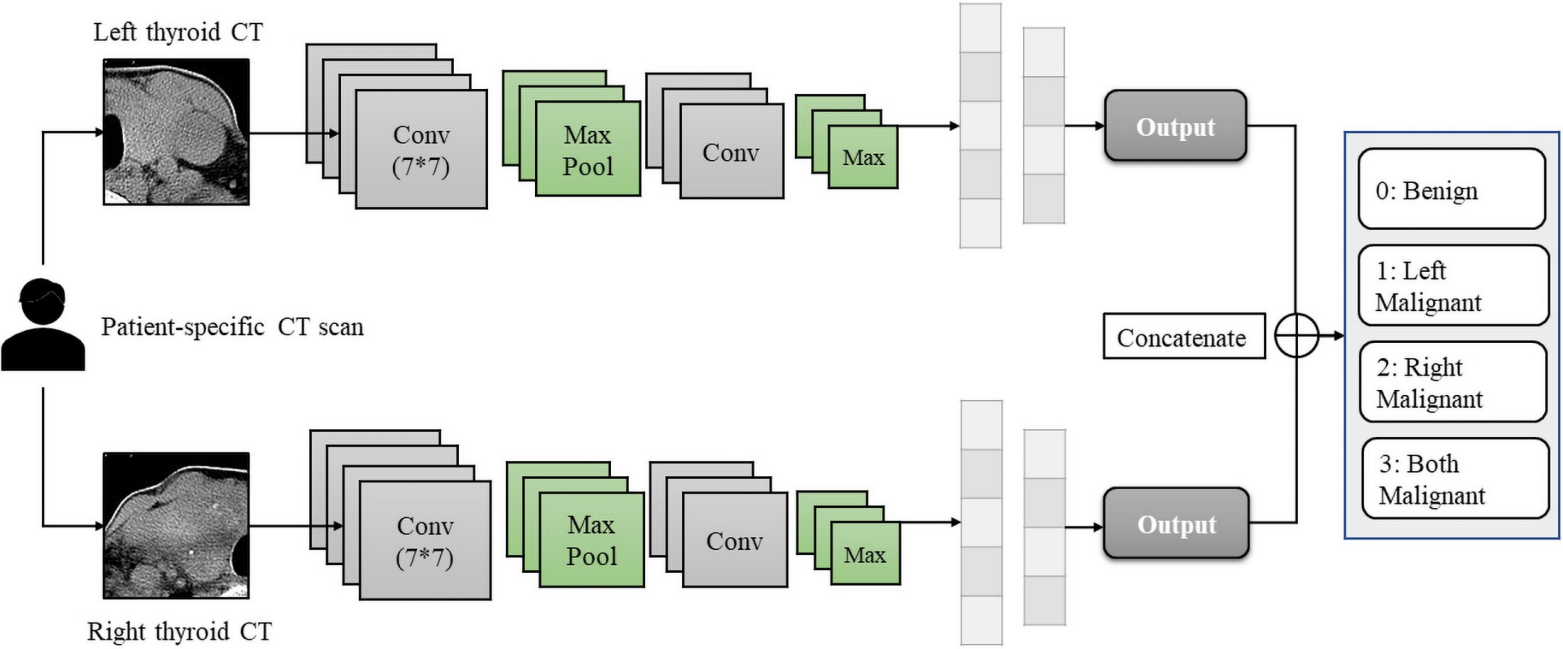
Double inputs dual-channel model.

Not only this research seeks to enhance thyroid diagnostic accuracy rates, but also expects to design a patient-specific malignant thyroid nodule detection model. Evidently, the SIDC model cannot achieve the goal since it focuses on cancerous lesion detection rather than diagnosing individual patient’s thyroid status. Hence, here proposes the second architecture shown in Fig 4: the Double Inputs Dual-Channel (DIDC) architecture.

Noteworthy, the DIDC architecture is made for CT scans solely at the current stage. The reason is that CT scans provide a comprehensive view of the thyroid gland, while ultrasound images focus on specific nodules. Looking at the overall presentation of the thyroid gland is much more convincing compare to paying attention to the individual nodule. Hence, CT scans were applied for the two patient-specific designs. Moreover, the proposed DIDC model has advantages in:

- It is designed as patient-specific to detect malignant thyroid nodules for individual patients.
- It offers the status of both sides of thyroid glands to the doctors and patients.
- The architecture outputs the overall status of the gland, making diagnostic and treatment decisions more efficiently.

However, applying this architecture has some prerequisites. CT scans provide a whole sight of thyroid glands, including the left and right lobes, while ultrasound images primarily include one or more nodules in each image at a time. In addition to that, not all patients undergo one type of thyroid disease at a time; sometimes, patients might have one side of the thyroid that appears to have cancerous nodules, while the other side remains normal. Personalized precision medicine is demanding in the medical domain as no two patients have 100% identical physical conditions or symptoms [76]; thus, feature maps generated from an individual patient or even individual lobes are distinguished from each other. Under this situation, segmenting whole thyroid CT glands into the left and right sides is necessary to make the labeling process much more accurate and efficient. As ultrasonography does not have this characteristic, this architecture was not implemented with ultrasound images.

Additionally, the left and right input images must be applied in two separate streams so the model can evaluate both sides simultaneously. Both left and right input CT scans must be extracted from one patient. Furthermore, the scale of both input data sets (i.e., the number of both input image sets) must be equal. Similarly, these requirements also apply to our four-channel architecture.

Fig 4 demonstrates the detailed architecture. A whole thyroid CT scan from an individual patient will be segmented into the left and right sides, and both data sets will need to be labeled. Each left and the right-side image will go through a single channel Xception model with the same filter size to obtain similar receptive fields for both sides. Then, images will travel through entry flow, middle flow, fully connected layers with softmax operator or logistic regression functions simultaneously. Further, both left and right-side outputs will be concatenated into a 4 × 4 matrix. Lastly, the model classifies the patient into either “benign” represented using 0, “left-side malignant” denoted as 1, “right-side malignant” using 2, and “both sides malignant” represented using 3.

#### Four-channel CNN model

Beyond the SIDC and DIDC models, we have extended to a four-channel CNN. Concerning the patient-specific design, our four-channel architecture retains both SIDC and DIDC’s characteristics. Concretely, the four-channel model has several advantages:

- It persists the increased accuracy rates obtained by the SIDC model since two different filter sizes were utilized.
- It is also designed as patient-specific obtained from the DIDC model.
- It enhances the original diagnostic accuracy, at the same time, helps doctors identify each patient’s thyroid status, and helps with decision-making more accurately and effectively.

**Fig 5:**
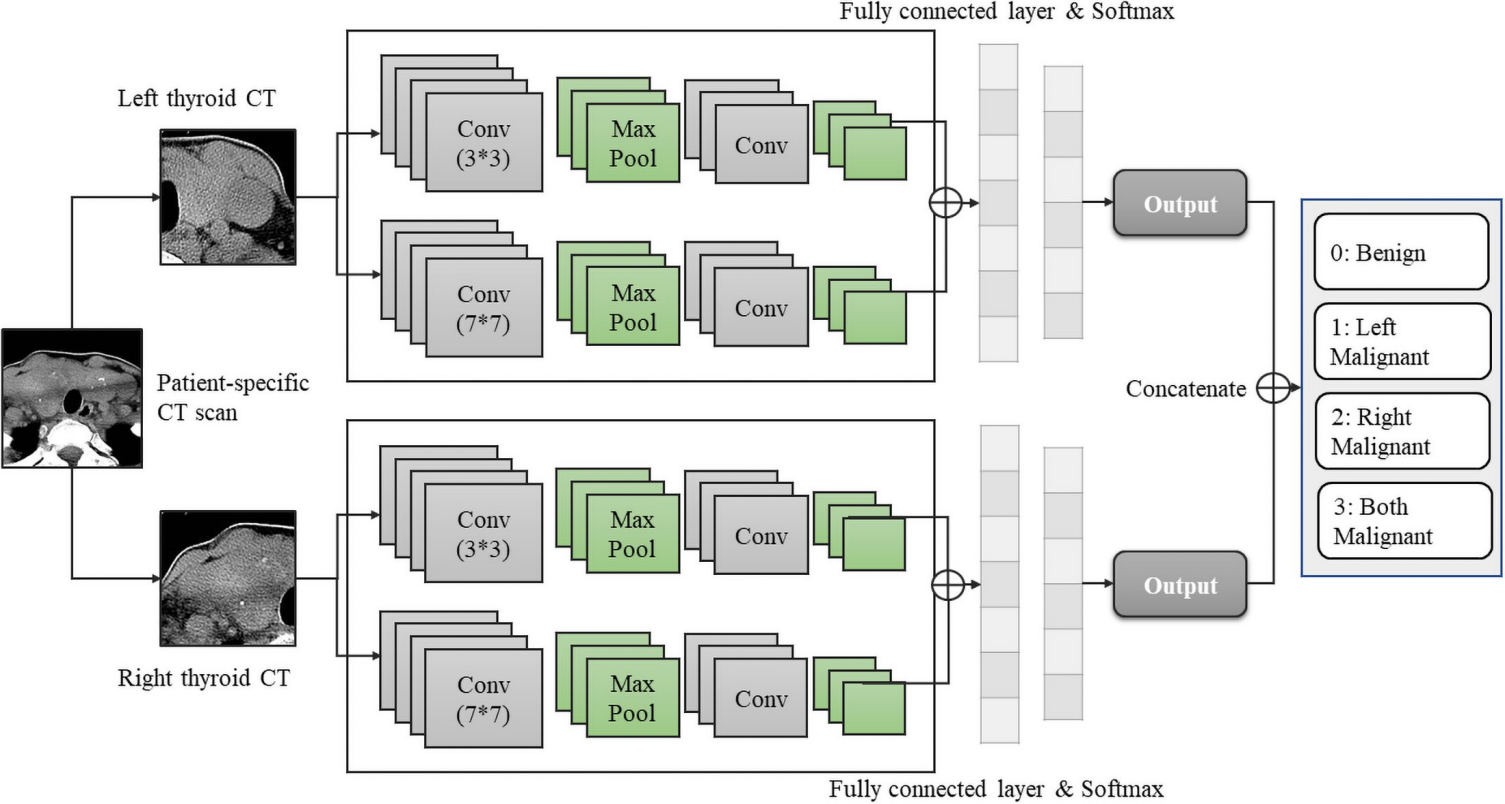
Double inputs dual-channel (four-channel) model.

The four-channel architecture in Fig 5 also only applies to CT scans. Specifically, the segmented left-side and right-side CT scans extracted from one patient will travel through two convolutional channels with different filter sizes, following a max-pooling layer as suggested in the Xception architecture. Again, the feature maps from two convolutional channels will be concatenated and sent to a fully connected layer, denoted using a vector indicating the probability of each class. And the two vectors will be further concatenated into a multi-class notations (same as DIDC where 0 represents “benign”, 1 represents “left-side malignant”, 2 represents “right-side malignant”, 3 denotes “both sides malignant”).

## Experiment settings

The MXTCD framework and the three proposed architectures were evaluated using real-world data sets, and the experimental settings are explained in this section. Given an input data set *X*, let $X_{i}\in R^{w\times h\times c}$, where *i* is the *i*th instance from *X*, and the corresponding image was denoted into *w* width and *h* height in *c* number of channel. In this study, all the experiments used the size of $X_{i}\in R^{224\times 224}$ for the input images, following the guidance made by [69]. Respectively, labels were set as *Y* ∈ {0, 1} for the binary classification task. Specifically, 0 represents “benign” thyroid nodules, including normal tissues, adenoma, and goiter, and 1 denotes “malignant” thyroid nodules, representing thyroid cancer.

### Data sets acquisition

We seek to demonstrate the impacts of different medical image modalities on diagnostic accuracy so that various medical imaging can be selected for future research implementation; thus, two types of medical image sets were adopted for this study.

#### Ultrasound images

This study is a comparative study, and two sets of ultrasound images were used to evaluate the proposed architectures.

*Open access ultrasound images*. We have utilized the Digital Database Thyroid Image (DDTI) for the ultrasound images, an open-access ultrasound images database offered by [77]. Experienced radiologists have classified the thyroid ultrasound images from DDTI following the Thyroid Imaging Reporting and Data System (TIRADS). Based on the TIRADS evaluations, there are seven categories of the images, including” (1) indicating normal, (2) is a benign nodule, (3) is for no suspicious ultrasound feature, (4a) is made for one suspicious ultrasound feature, (4b) two suspicious ultrasound features, (4c) three or four suspicious ultrasound features and (5) five or more suspicious ultrasound features [77].

**Fig 6:**
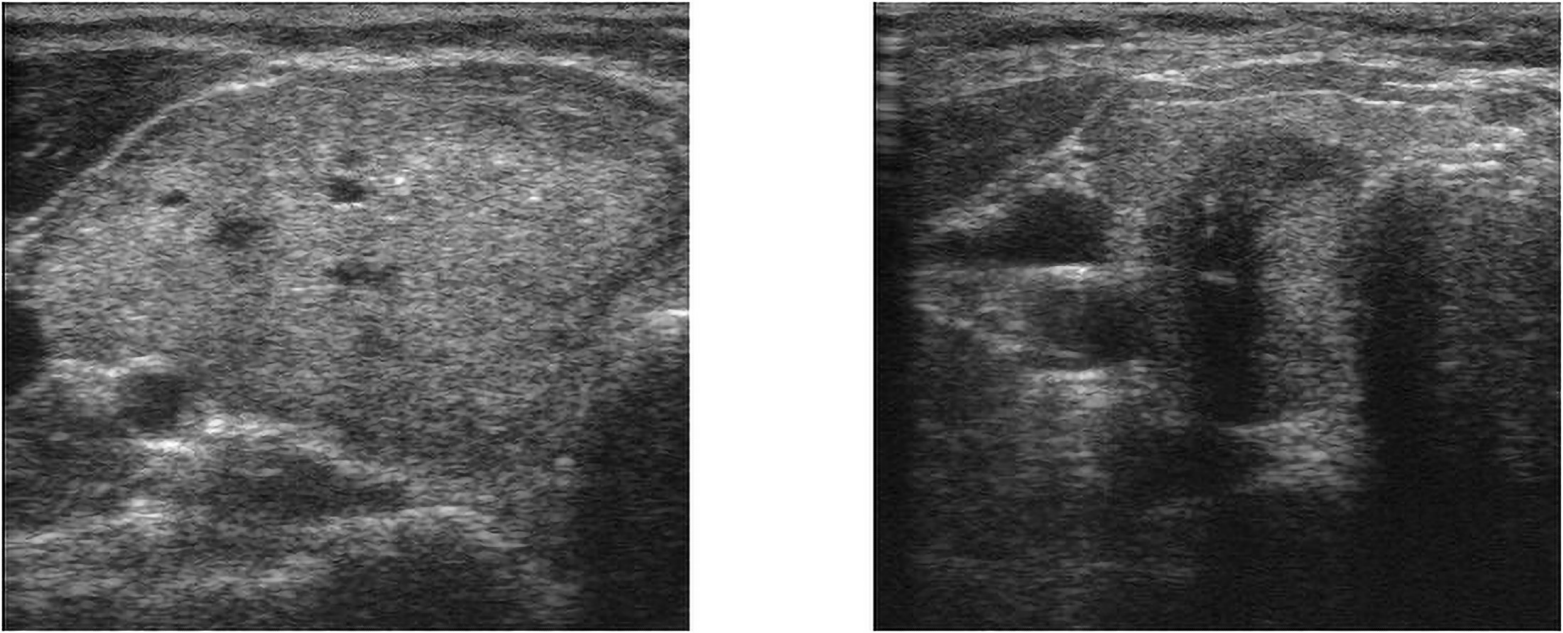
Ultrasound images from DDTI: TIRADS 2 on the left-side with well defined margins and no calcification, labeled as “benign”; TIRADS 5 on the right-side with micro-calcification, labeled as “malignant”.

To align with previous studies [6, 78], we have selected our benign images with TIRADS ranking 1, 2, and 3, while the left 4*a*, 4*b*, 4*c*, and 5 were labeled as malignant. In this case, a total of 448 thyroid ultrasound images were selected. Among them, 66 images were labeled as benign, and 382 were labeled as malignant. Fig 6 gives an example of our selected benign and malignant images.

*Hospital ultrasound images*. Besides the DDTI images, we have also obtained our research data sets from a first-class hospital (Hospital_X) developed the earliest in the northern side of Sichuan province in China, with more than 30 departments, and its thyroid department was founded several years ago yet received and cured more than 10, 000 patients up to now. With the ethics approval obtained from Monash Human Ethics Committee, we have collected 917 ultrasound images in Hospital_X. Among the 917 ultrasound images, 200 of them were labeled as benign, and 717 images were labeled as malignant.

#### Hospital CT scans

CT scans can detect abnormal thyroid glands based on their shapes and densities [79]; after that, they obtain much more information than ultrasound images when applying automated machine learning approaches for diagnosis. Furthermore, for ultrasound images, there is no point in differentiating left or right-sided thyroid nodules since each nodule would be classified based on their shapes, solid texture, and margins [80].

**Fig 7:**
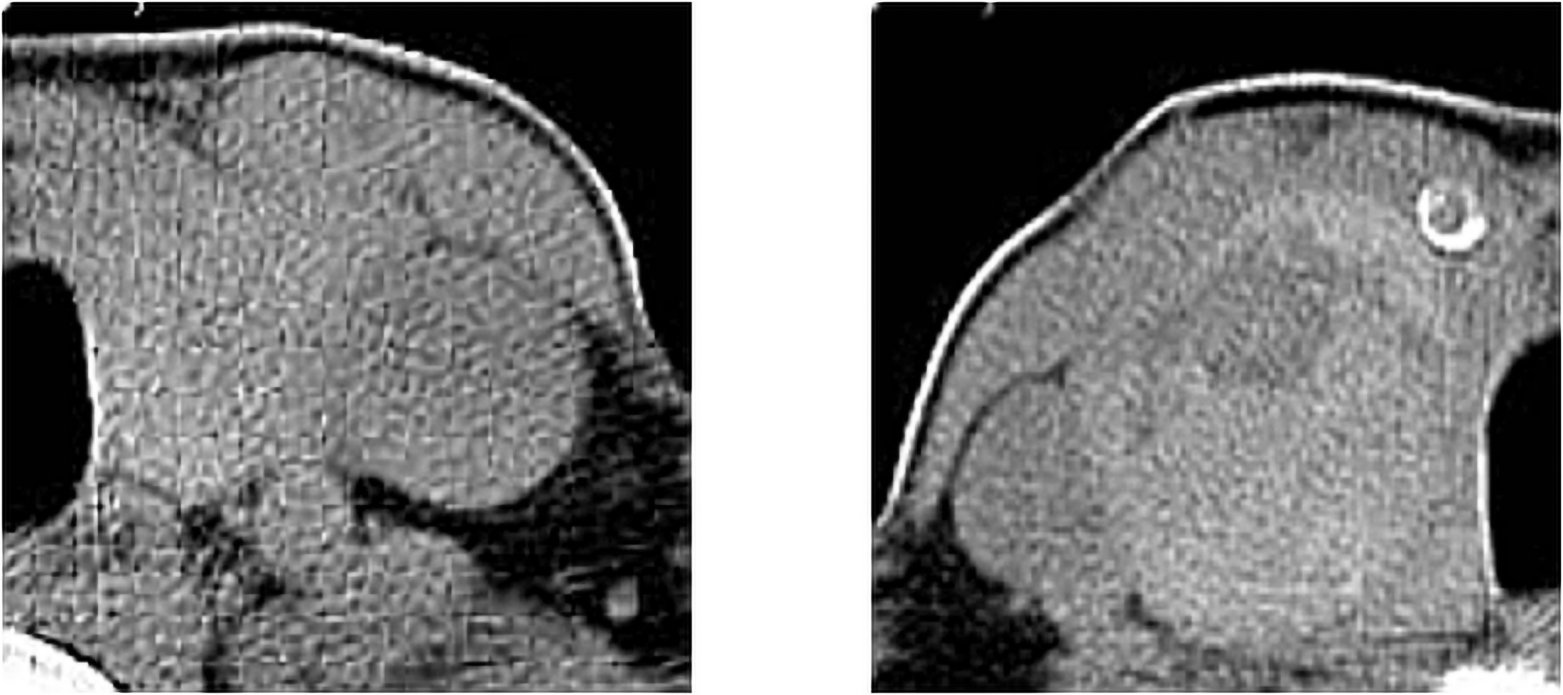
Segmented thyroid gland CT scans from patient No. 277: Left-side is Goiter (Benign) and right-side is Papillary Cancer (Malignant).

On the contrary, when detecting abnormal thyroid nodules using CT scans, separating the whole images into left and right-sided is necessary because not all the patients would only undergo one type of thyroid disease for both sides. In this case, cutting the whole thyroid CT scans into the left and right sides helps train the CNNs more efficiently and accurately. Besides, patients and doctors can have a clearer view of the thyroid’s abnormal side and make decisions correspondingly. Fig 7 demonstrates the segmented thyroid CT scans.

Overall, in this research study, from Hospital_X, we also collected 2, 352 thyroid CT scans. We have included 1, 224 left-side thyroid scans containing 257 benign and 967 malignant thyroid nodules. The 1, 128 right-side scans contain 257 benign and 871 malignant thyroid nodules. Table 1 demonstrates the details of the two sources, along with both ultrasound and CT images details.

**Table 1:** Data sets descriptions.

Image Type	Source	Patient	Class		Total
			Benign	Malignant	
Ultrasound	DDTI	400	66	382	448
	Hospital_X	578	200	717	917
CT	Hospital_X	578	514	1838	2352

In this work, Hospital_X provided the de-identified data including medical records, ultrasound and CT scans, diagnostic reports, procedures, and test results. The personal information was removed from all these data by the hospital before sharing.

Furthermore, in the medical image analysis field, an issue on data set imbalanced is common. Most people were suffering from malignant thyroid nodules not knowing, that is because thyroid cancer is painless, and malignant thyroid nodules are hard to be noticed. When they have symptoms like hard to breathe, difficulty swallowing, appearing to have a husky voice, and others, they usually already have malignant thyroid nodules. At that time, most people go to hospitals and are asked to perform radiological examinations, resulting in more malignant cases. While in the machine learning field, we have different ways to handle imbalanced image sets, including mirroring, rotating, cropping, and flipping images, to name a few. Our research focuses on medical images with similar structures and textures for the same type of images, and we would like to maintain the images’ original features. Therefore, the typical image augmentation approaches are not appropriate. To handle the imbalance of the data set, we have adopted an efficient training-testing split technique to ensure that the dataset can be divided into training and testing sets with the same proportion of observations for the two classes. We believe, through this approach, the input data set can be imbalanced, and clinicians and researchers can skip the data augmentation stage; meanwhile, the testing accuracy maintains fairness.

### Parameters settings

The MXTCD framework was implemented as follows. In the initial pre-processing stage, each DDTI ultrasound image was labeled based on its TIRADS scores, hospital ultrasound images, and the CT scans were labeled according to their histopathological results; otherwise, images were eliminated from this research. We have designed a thyroid image segmentation tool in Python (also available on GitHub) to efficiently crop the medical images and give labels accordingly to be fed into CNNs directly.

Overall, 448 open access ultrasound images were acquired from 400 patients. 917 hospital ultrasound images were obtained from 578 patients. 2, 929 CT scans were obtained from the same patients, while 577 images were non-diagnostic and were removed. This study, therefore, adopted the 2, 352 CT scans, with 1, 224 left-side and 1, 128 right-side were separately split into training and testing sets. All the images (both ultrasound and CT images) are pre-processed so that they are in the same size with 224 × 224 pixels.

Medical images are complex to process, and in reality, they are always imbalanced. Therefore, we have adopted a 10-fold stratified cross-validation rather than a typical cross-validation approach to get a fair result for dealing with imbalanced data sets, that is, to obtain the best ratio of the benign and malignant class in both training and testing sets. Specifically, the stratified cross-validation algorithm allows the training and testing split to retain each class’s ratio where each fold has the same proportion of observations with a given category; this allows the variance of the estimates to be reduced [81]. It compensates for the unequal number of image types within each label and the uneven distribution among the classifications; thus, the classification results can be much more representative.

Additionally, the computational resources utilized for all the experiments are Windows 10 Pro with 64-bit 16 gigabytes memory with an AMD Ryzen 5 2600 Six-Core processor, and a GeForce GTX 1050 Ti GPU.

As for the hyper-parameters settings, the filter size for dual- and four-channel were tested using 3 × 3 and 7 × 7 since they are the typical kernel sizes for CNNs. Additionally, we have adopted the Adam optimizer, and the learning rate was gradually updated during the optimization process because many models started to get vanishing gradients with an initial learning rate of 1 × 10^−2^. Lastly, the best performing learning rate was found to be 1 × 10^−5^ for the evaluated models. Moreover, in each model, the batch size was set to 5 during the training process.

Moreover, the cost function we have utilized is the binary cross-entropy shown in Eq 4. In this case, *g* and (1 − *g*) are the ground truth label for each image, and *y* and (1 − *y*) are the predicted class for the image.

$$\begin{array}{c} BCE=-glog(y)-(1-g)log(1-y) \end{array}$$ (4)

With the stratified 10-fold cross-validation, the test accuracy was recorded in the meantime. Precisely, the best testing accuracy, *f*1 score, precision (also is positive predictive value), negative predictive value (npv), and recall were calculated for each fold. In general, we have compared the 11 models based on their average testing accuracy, precision, recall, npv, and *f*1-score from the 10 − *fold* cross-validation through Eqs 5 to 9. Here, we have calculated them based on their confusion matrix. *k* is number of folds, *TP* stands for “True Positive”, *TN* stands for “True Negative”, *FP* stands for “False Positive”, and *FN* stands for “False Negative”.

$$\begin{array}{c} Accuracy=\frac{1}{k}\sum_{i=1}^{k}\frac{TP_{i}+TN_{i}}{TP_{i}+TN_{i}+FP_{i}+FN_{i}} \end{array}$$ (5)

$$\begin{array}{c} Precision\left(PPV\right)=\frac{1}{k}\sum_{i=1}^{k}\frac{TP_{i}}{TP_{i}+FP_{i}} \end{array}$$ (6)

$$\begin{array}{c} NPV=\frac{1}{k}\sum_{i=1}^{k}\frac{TN_{i}}{TN_{i}+FN_{i}} \end{array}$$ (7)

$$\begin{array}{c} Recall=\frac{1}{k}\sum_{i=1}^{k}\frac{TP_{i}}{TP_{i}+FN_{i}} \end{array}$$ (8)

$$\begin{array}{c} F1=2\times \frac{Recall\times Precision}{Recall+Precision} \end{array}$$ (9)

With the initial training and testing through the Xception model using all the image sources, we have established a baseline result with the single-channel architecture to compare to our developed multi-channel architectures. Besides, the SIDC architecture was applied on both ultrasound and CT images to enhance its classification results. For the DIDC and four-channel models, only CT scans were adopted to meet the patient-specific goal. Therefore, we have to obtain the same amount of left-side and right-side CT scans from a specific patient for two input streams. In general, 965 left-side and 965 right-side CT images were extracted from 209 patients. For the left side, there are 172 benign and 793 malignant scans. For the right side, there are 200 benign and 765 malignant scans. Furthermore, we have maintained the 10-fold stratified cross-validation approach with Adam optimizer and 1 × 10^−5^ learning rate for the evaluations.

## Results analysis

We have conducted a group of experiments to compare existing techniques with our choice of neural network (Xception). Also, we present that the choice of medical images and image sources play critical roles in thyroid cancer detection tasks. The results are explained in this section.

### Binary classification results

Initially, we have applied the Xception model and compared its result with other 10 models through binary classification tasks using both ultrasound images and CT scans. This allows us to evaluate our choice of CNN selection through ablation experiments. This result is presented as the baseline result. All the models were compared based on their classification accuracy rates, precision, npv, recall, *f*1 scores, and running time.

#### Ultrasound images binary experimental results

Table 2 provides the detailed experimental results for thyroid cancer detection tasks with ultrasound images. It indicates that for the DDTI sets, Xception has reached the testing accuracy of 0.980, precision of 0.99, 1.0 for npv, 0.945 for recall, and 0.967 for *f*1 score. The hospital images have gained an accuracy of 0.987, precision of 0.985, npv of 0.99, recall of 0.975, and *f*1 of 0.98 through Xception. There is no doubt that Xception outperforms all the other models. While DenseNet121 is the second-best performing model with an accuracy of 0.978 for DDTI images and 0.965 for hospital images.

**Table 2:** Comparison of various CNN models in binary classification tasks: Different sources of ultrasound images were applied for thyroid cancer detection.

DDTI							
Model	Accuracy	Precision	NPV	Recall	F1	No. parameters	Time (min)
VGG8	0.857	0.830	0.667	0.860	0.845	5, 516, 610	46
VGG11	0.832	0.830	0.156	0.635	0.720	10, 826, 306	58
VGG16	0.853	0.730	0.136	0.850	0.785	16, 320, 514	118
VGG19	0.783	0.853	0.156	0.901	0.876	21, 630, 210	141
ResNet10	0.864	0.880	1.0	0.860	0.870	4, 912, 578	33
ResNet18	0.873	0.870	0.846	0.870	0.870	11, 187, 138	53
ResNet50	0.850	0.730	0.852	0.850	0.785	23, 591, 810	82
DenseNet121	0.978	0.985	1.0	0.925	0.954	7, 039, 554	149
Xception	**0.980**	**0.990**	**1.0**	**0.945**	**0.967**	20, 865, 578	131
InceptionV3	0.967	0.980	1.0	0.885	0.930	21, 806, 882	117
InceptionResNetV2	0.971	0.985	1.0	0.900	0.941	54, 339, 810	182
**Hospital**							
VGG8	0.799	0.790	0.802	0.800	0.795	5, 516, 610	94
VGG11	0.815	0.688	0.827	0.275	0.393	10, 826, 306	142
VGG16	0.809	0.800	0.814	0.810	0.805	16, 320, 514	241
VGG19	0.792	0.680	0.795	0.090	0.151	21, 630, 210	297
ResNet10	0.832	0.830	0.828	0.830	0.830	4, 912, 578	46
ResNet18	0.828	0.830	0.828	0.830	0.830	11, 187, 138	109
ResNet50	0.865	0.870	0.861	0.860	0.865	23, 591, 810	218
DenseNet121	0.965	0.965	0.965	0.930	0.947	7, 039, 554	203
Xception	**0.987**	**0.985**	**0.990**	**0.975**	**0.980**	20, 865, 578	214
InceptionV3	0.924	0.925	0.928	0.840	0.880	21, 806, 882	241
InceptionResNetV2	0.957	0.982	0.952	0.820	0.894	54, 339, 810	377

#### CT scans binary experimental results

Similarly, in order to compare the choice of medical images, we have also inputted CT scans for baseline evaluations, and Table 3 presents the results. Unsurprisingly, the Xception model again outperforms other models using CT scans. With the left-side CT scans, Xception has reached an accuracy rate of 0.966, precision of 0.961, npv of 0.986, recall of 0.997, and an *f*1 score of 0.979. As for the right-side, Xception has achieved an accuracy of 0.970, 0.967 of precision, 0.983 of npv, recall of 0.995, and an *f*1 score of 0.981. DenseNet121 is again the second-best performing CNN model, and it reached accuracy with 0.954 on the left-side CT scans and 0.940 on the right-side CT scans.

**Table 3:** Comparison of different CNNs on thyroid cancer diagnosis via CT.

Model	Accuracy		Precision		NPV		Recall		F1		Time (min)	
	Left	Right	Left	Right	Left	Right	Left	Right	Left	Right	Left	Right
VGG8	0.801	0.785	0.807	0.788	0.659	0.707	0.984	0.986	0.887	0.876	130	106
VGG11	0.687	0.738	0.807	0.802	0.268	0.389	0.794	0.877	0.800	0.838	159	143
VGG16	0.798	0.669	0.800	0.787	0.727	0.277	0.994	0.784	0.886	0.786	275	270
VGG19	0.678	0.777	0.796	0.780	0.233	0.667	0.796	0.992	0.796	0.873	321	297
ResNet10	0.815	0.774	0.845	0.822	0.603	0.506	0.938	0.904	0.889	0.861	52	48
ResNet18	0.812	0.780	0.828	0.810	0.634	0.537	0.962	0.935	0.890	0.868	81	74
ResNet50	0.895	0.878	0.902	0.886	0.860	0.832	0.974	0.966	0.936	0.924	221	203
DenseNet121	0.954	0.940	0.955	0.938	0.953	0.948	0.989	0.987	0.972	0.962	281	260
Xception	**0.966**	**0.970**	**0.961**	**0.967**	**0.986**	**0.983**	**0.997**	**0.995**	**0.979**	**0.981**	263	240
InceptionV3	0.914	0.895	0.927	0.914	0.855	0.817	0.968	0.954	0.947	0.934	207	205
InceptionResNetV2	0.892	0.872	0.906	0.877	0.821	0.847	0.964	0.971	0.934	0.922	510	417

**Fig 8:**
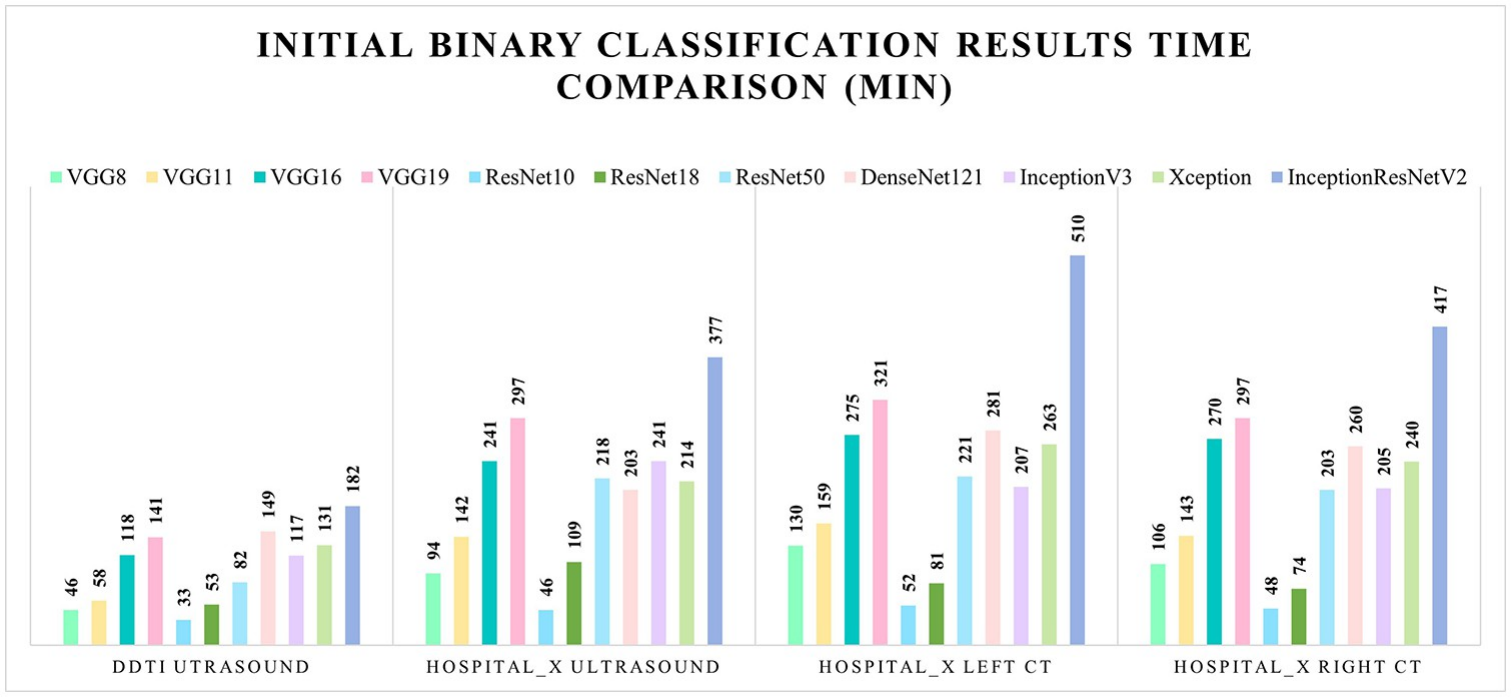
Initial binary classification task running time comparison for the 11 models.

Besides the pre-trained binary classification results, all the models’ running time was also demonstrated in Fig 8 where ResNet10 obtains the fast running time, Xception reached a similar running time as DenseNet 121.

Accordingly, we also applied both ultrasound data sets through our SIDC model to enhance its diagnostic accuracy rates. As for DIDC and four-channel architectures, only CT scans were implemented.

### Single input dual-channel CNN results

To select an appropriate filter size for the SIDC model, we have tested two single channels (when filter size = 3 × 3, and when filter size = 7 × 7) for both ultrasound images and CT scans. Besides, the combination of two kernel sizes was implemented as our SIDC model, and their results’ comparison is presented in Table 4.

**Table 4:** Single-channel and dual-channel comparison.

Data set	Architecture	Filter	Accuracy
DDTI Ultrasound	Single	3	0.980 (±0.003)
	Single	7	0.984 (±0.018)
	SIDC	3, 7	0.987 (±0.001)
Hospital_X Ultrasound	Single	3	0.987 (±0.002)
	Single	7	0.988 (±0.003)
	SIDC	3, 7	0.989 (±0.003)
Hospital_X Left CT	Single	3	0.966 (±0.004)
	Single	7	0.972 (±0.007)
	SIDC	3, 7	0.975 (±0.008)
Hospital_X Right CT	Single	3	0.970 (±0.004)
	Single	7	0.974 (±0.013)
	SIDC	3, 7	0.975 (±0.005)

For both ultrasound images and CT scans regarding the binary classification results, the Xception model performs better when filter size equals 7 compare to the original embedded filter size of 3. When the filter size changed to 7, the model reached an accuracy of 0.984 for DDTI ultrasound images, 0.988 for hospital ultrasound images, 0.972 on the left-side CT data set, and 0.974 on the right-side CT data set.

When applying the SIDC architecture, there presents minor increases in testing results with both medical image types. For the DDTI set, the accuracy increased from 0.984 to 0.987. For hospital_X ultrasound images, the accuracy increased from 0.988 to 0.989. For CT images, increases can be found in both left-side and right-side CT scans. The SIDC model architecture increased the accuracy to 0.975 for both left-side and right-side CT scans. Comparing to the original single-channel Xception, the accuracy increased from 0.980 to 0.987 for DDTI ultrasound, 0.987 to 0.989 for hospital_X ultrasound, 0.966 to 0.975 for left-side CT, and 0.970 to 0.975 for right-side CT scans.

### Double inputs dual-channel results

The SIDC architecture proves to reach promising increased diagnostic results when detecting malignant thyroid nodules. Further, we proposed the double-input streams with dual-channel architecture. It can detect malignant thyroid nodules for individual patients, called the “patient-specific thyroid cancer detection architecture”. As explained in Section, this architecture’s result demonstrates the input patient’s thyroid status. And each of the four classes (class 0 to 3) were examined based on their classification precision, recall, and *f*1 scores (see Table 5).

**Table 5:** DIDC and four-channel comparison. Class 0 to 3 indicates the patient either has normal thyroid (0), has malignant left-side thyroid (1), has right-side thyroid malignant (2), or has both sides malignant (3).

Architecture	Class	Precision	Recall	F1
DIDC	0	0.97 (±0.091)	0.86 (±0.091)	0.91 (±0.091)
	1	0.99 (±0.088)	0.94 (±0.089)	0.96 (±0.089)
	2	0.97 (±0.089)	0.88 (±0.090)	0.92 (±0.090)
	3	0.96 (±0.006)	0.99 (±0.000)	0.97 (±0.002)
Four-channel	0	1.00 (±0.090)	0.78 (±0.110)	0.88 (±0.096)
	1	0.97 (±0.087)	0.91 (±0.089)	0.94 (±0.086)
	2	0.94 (±0.099)	0.89 (±0.089)	0.91 (±0.093)
	3	0.95 (±0.007)	0.99 (±0.001)	0.97 (±0.002)

### Four-channel results

With the four-channel architecture, this research study seeks to offer individual patients the enhanced diagnostic accuracy rates. Again, the same as the DIDC model, the four-channel model’s output also presents that each class was examined based on precision, recall, and *f*1 scores. Table 5 presents the results for DIDC and four-channel architectures.

**Fig 9:**
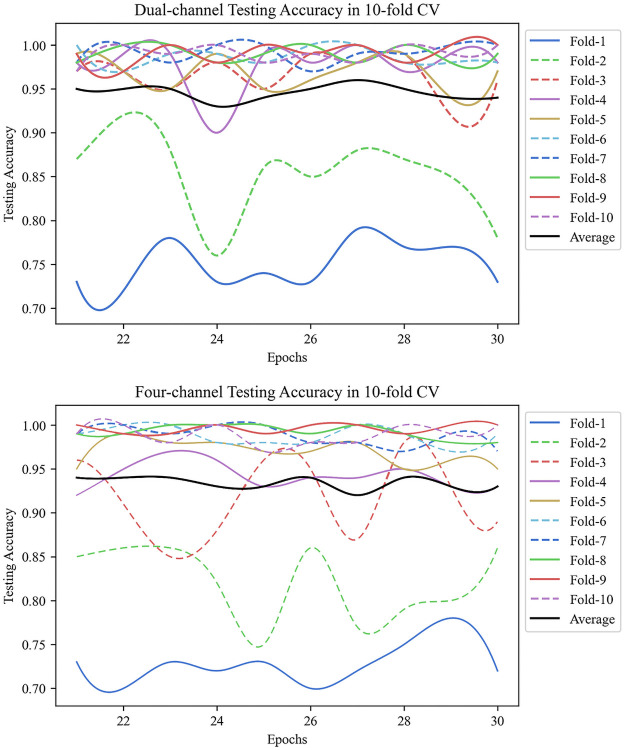
DIDC and four-channel 10-fold cross-validation accuracy results. The average 10-fold scores for each epochs were demonstrated using the black line, and the average testing accuracy for DIDC is 0.95, for four-channel is 0.94.

Fig 9 suggests that imbalanced data sets would result in some fluctuations from the 10-fold cross-validation results. Other than these fluctuations, our model is relatively stable and performs promising. The best performing results from each fold were averaged for the 10-folds, and their averaged results can be found in Table 5.

Overall, both architectures obtained similar diagnostic results where the DIDC model reached an average accuracy of 0.95, and the four-channel achieved an average accuracy of 0.94. Comparing the four classes regarding their precision scores, the four-channel model has achieved a 1.00 accuracy for detecting “normal” patients, outperforming the DIDC model. While when detecting abnormal patients, the DIDC model is slightly better than the four-channel architecture. Specifically, DIDC model obtained 0.97 accuracy score for diagnosing “normal” patient, 0.99 for “left-side abnormal”, 0.97 for “right-side abnormal”, and 0.96 for “both sides abnormal”. Those four classes were scored 1.00, 0.97, 0.94, and 0.95 for the four-channel architecture.

## Discussion

Thyroid disease needs to be early detected to achieve low morbidity and mortality rates. Currently, thyroid disease diagnosis proceeds manually through medical images, blood examinations, FNAC, and excisional biopsies, which is inefficient, time-consuming, and might be affected by unreliable human false-positive rates. Machine learning has become a powerful tool for improving disease diagnosis. Existing studies applying ultrasound images to distinguish abnormal thyroid nodules is inefficient, as they require clinicians to determine the region of interest, following feature extraction and selection to classify nodules one by one [19]. Besides, with the development of deep learning, advanced models were designed yet tested on thyroid cancer detection tasks. Hence, we have developed an adaptable framework that consists of three optional multi-channel CNN architectures with the Xception model as the base structure. All three architectures were evaluated using real-world data sets and proven to outperform existing studies on thyroid cancer diagnosis. Additionally, the choice of outputs (i.e., binary and multi-class) leads to the highly interpretable results that generate the potential adoptions in clinical.

### Ultrasound

Most existing studies can reach a diagnostic accuracy rate of 70% to 92% using ultrasound images [19, 20, 57, 70, 82]. Our work has increased the diagnostic accuracy for thyroid cancer detection, where our base model Xception has reached an accuracy of 0.980 in the DDTI data sets and 0.987 in the Hospital_X ultrasound images. In addition, Xception and its updated multi-channel architectures have evidently outperformed the other conventional single-channel CNN models. Specifically, the SIDC model obtained a diagnostic accuracy of 0.987 for the DDTI ultrasound images and 0.989 for the hospital ultrasound images. The results indicate that filter size is influential to CNN performance, adding channel numbers into CNN models can increase its performance. In addition, comparative studies on different medical imaging modalities are also absent in the existing work. When comparing the diagnostic accuracy for CT scans and ultrasound images in this study, it is quite engaging that ultrasound performs slightly better than CT scans; this might be related to the characteristics of ultrasonography. Ultrasound is highly sensitive to human intervention as it is performed based on radiologists’ experience. Thus, during the image acquisition process, radiologists usually select the nodules that appear to have apparent features for diagnosing; this might be a potential cause of the higher diagnostic accuracy of ultrasound than CT, as CT is a fully automated process where the chance of human intervention is much lower.

### CT

Among all the existing studies for thyroid cancer detection with CAD adoption, the utilization of CT scans is significantly limited. The work conducted by [83] adopted CT scans through CNN for detecting thyroid cancer metastasis, and their work reached an accuracy score of 0.904. Moreover, [84] fused two CNNs for CT scans to detect malignant thyroid nodules and obtained an accuracy of 95.73. While in this study, we obtained an accuracy of 0.966 for the hospital left-side CT scans and 0.970 for the right-side CT scans through the base model. Moreover, we have reached 0.975 classification accuracy rates for both left and right-side input thyroid CT images with the SIDC architecture. These results are superior to current existing works. Additionally, the patient-specific DIDC and four-channel models are the novelty of this paper and do not have any benchmark results to compare. These two models also demonstrate outstanding classification results where DIDC has achieved 0.95 diagnostic average accuracy, and the four-channel model has obtained a 0.94 average accuracy score. Besides, CT scans can reach comparable detection results comparing to ultrasound images; this highlights the potential adoption of other medical image modalities for the implementation of CAD approaches. Comparison details with existing works can be viewed in Table 6.

**Table 6:** Experiments comparison with existing literature.

Image Modality	Methods	Architecture	Image	Accuracy
Ultrasound	[50]	ResNet-18	DDTI	0.840
	[78]	VGG-16+SVM	DDTI	0.940
	[85]	EQP+HOS+PSO+SVM	DDTI	0.970
	**Proposed**	**SIDC**	**DDTI**	**0.987**
CT	[86]	Unet+CNN-F	832 scans	0.859
	[83]	ResNet-50	995 scans	0.904
	[84]	CNN-F	1421 scans	0.957
	**Proposed**	**SIDC**	**2352 scans**	**0.975**

The overall testing results for three architectures highlight the potential application of a patient-centric design; thus, each patient can be diagnosed more efficiently in the near future. Moreover, the three architectures’ outputs are highly interpretative since doctors can understand a patient’s thyroid status through the outputs directly. In order to better interpret the model and the results, we tend to comply with the Shapley values theory [87], which was not explicitly mentioned in this research as it is beyond the research scope.

## Conclusion

To conclude, we have proposed a thyroid cancer detection framework through the Xception model, and our model was evaluated from three different architectures using different medical images. Additionally, our model was compared with existing algorithms and existing literature studies. Overall, this research study makes contributions from the following four aspects.

- **Potential application for patient-centric design in the clinical field**: The proposed MXTCD framework incorporates three different multi-channel CNN architectures designed for the patient-centric purpose. This framework has high interpretability that allows clinicians to obtain the enhanced diagnostic accuracy. Therefore, it leads to the potential adoption in the clinical field regarding machine learning-driven approaches for diagnosis.
- **Increased diagnostic accuracy and efficiency with comparative analysis**: Through the framework, the malignant thyroid nodule diagnostic accuracy has been significantly improved. With the evaluated 11 models, we have provided a comprehensive experimental results comparison, suggesting that our choice of the neural network, Xception, outperforms most of the CNNs under this circumstance; also, it generalises well to different medical image types. Additionally, we have compared different data sources and medical image modalities in this work, which indicates that the thyroid cancer diagnosis has improved regarding its accuracy and efficiency.
- **Potential adoption of various medical imaging**: Currently, a large number of researches focus on applying CAD with ultrasound images. With our research, the framework was evaluated using different medical images. Limited studies have adopted CT images for classifying thyroid nodules, and this paper contributes to the adoption of CT images, proving the application of CT images is as precise and efficient as ultrasound images. This can be further broadcast to the community to enlarge the way we conduct research, since it is proven that different medical images can be adopted for the CAD system to achieve increased diagnostic results.
- **Application of multi-channel CNNs**: This paper demonstrates that multi-channel architecture is firstly introduced in the thyroid cancer detection area, and it has been proven to gain increased diagnostic accuracy rates compared to single-channel CNNs. Therefore, the application of this adaptable architecture is expected to be broadcast.
- **Society benefits**: With the increased diagnostic accuracy and efficiency, patients can be released from mental and financial pressure brought by the current clinical diagnostic procedures, and correspondingly, clinicians can make decisions more effectively regarding thyroidectomy. Thus, the early detection of thyroid disease can be achieved, leading to lower mortality and morbidity rates.

This work has several limitations. In reality, the balanced data set is relatively rare. From the precision and accuracy scores obtained for three architectures, we can see that our framework can outperform existing studies while concerning the npv and recall scores, our approach may have limitations regarding the imbalanced data set. Hence, we will test our framework on a more extensive balanced data set in future work.

Additionally, this paper adopts two ultrasound image sources for comparison, yet the CT images were relatively unitary. Therefore, in future work, we would like to adopt different CT scan sources for making comparisons. Besides, our study proves that various medical imaging can be adopted for thyroid cancer diagnosis. Hence, we will implement other types of medical images for diagnosis in our future work.

Further, we expect to get our hands on supporting doctors to classify thyroid cancer types through the architectures. Ideally, we expect to design a patient-centric thyroid cancer classification model where an individual patient can be examined among the six thyroid nodule types (goiter, adenoma, papillary carcinoma, follicular carcinoma, anaplastic carcinoma, and medullary carcinoma).
